# Clinical Benefit of First-Pass Recanalization Is Time-Dependent in Endovascular Treatment of Acute Ischemic Stroke

**DOI:** 10.3390/jcm12206596

**Published:** 2023-10-18

**Authors:** Jang-Hyun Baek, Ji Hoe Heo, Hyo Suk Nam, Byung Moon Kim, Dong Joon Kim, Young Dae Kim

**Affiliations:** 1 Department of Neurology, Kangbuk Samsung Hospital, Sungkyunkwan University School of Medicine, Seoul 03181, Republic of Korea; janghyun.baek@gmail.com; 2 Department of Neurology, Severance Stroke Center, Severance Hospital, Yonsei University College of Medicine, Seoul 03722, Republic of Korea; jhheo@yuhs.ac (J.H.H.); hsnam@yuhs.ac (H.S.N.); 3 Interventional Neuroradiology, Department of Radiology, Severance Stroke Center, Severance Hospital, Yonsei University College of Medicine, Seoul 03722, Republic of Korea; bmoon21@hanmail.net (B.M.K.); djkimmd@yuhs.ac (D.J.K.)

**Keywords:** first-pass recanalization, endovascular treatment, thrombectomy, stroke, outcome

## Abstract

Clinical benefit can be time-dependent even after first-pass recanalization (FPR) in endovascular treatment of acute stroke. This study aimed to evaluate the association between favorable outcome and FPR under a specific time frame. Patients who underwent mechanical thrombectomy were retrospectively reviewed. Recanalization status was categorized into four groups based on FPR and dichotomized time from groin puncture to recanalization (P-to-R time). Favorable outcomes were compared between groups. A total of 458 patients were included. As the cutoff of P-to-R time for favorable outcome was 30 min, recanalization status was categorized into FPR (+) with a P-to-R time ≤ 30 min (Group 1), FPR (–) with a P-to-R time ≤ 30 min (Group 2), FPR (+) with a P-to-R time > 30 min (Group 3), and FPR (–) with a P-to-R time > 30 min (Group 4). Favorable outcomes in Group 3 (37.5%) were significantly less frequent than those in Group 1 (60.4%, *p* = 0.029) and Group 2 (59.5%, *p* = 0.033) but were not significantly different from those in Group 4 (35.7%, *p* = 0.903). Compared to Group 1, Group 3 (adjusted odds ratio, 0.30 [95% confidence interval, 0.12–0.76]; *p* = 0.011) and Group 4 (0.25 [0.14–0.48]; *p* < 0.001) were adversely associated with favorable outcomes. FPR was associated with functional outcome in a time-dependent manner. Even for patients who have achieved FPR, their functional outcome might not be favorable if the P-to-R time is >30 min.

## 1. Introduction

Achieving significant recanalization is an ultimate goal of endovascular treatment of acute ischemic stroke [1]. A higher degree of revascularization is a well-known prognostic factor of an endovascular treatment [2,3]. In fact, modified Thrombolysis In Cerebral Infarction (mTICI) grade 2b or 3 has long been proposed as a common technical goal of an endovascular treatment [1,2]. However, more rigorous conditions of recanalization have been recently highlighted for the best endovascular performance. First, beyond a traditional recommendation of mTICI grade 2b or 3, extended TICI (eTICI) grade 2c or 3 (near-complete or complete) revascularization is associated with consistent and better functional outcomes after endovascular treatment [3,4,5,6]. Second, recanalization only by a single pass of a thrombectomy device has been suggested as a surrogate for rapid recanalization [7,8]. Based on these concepts, many recent studies have adopted first-pass recanalization (FPR) as a principal endovascular outcome [9,10,11].

However, not all FPRs are beneficial. Although a single pass in FPR is a surrogate for rapid recanalization, FPR cannot be always rapid. For example, a single pass of FPR can be delayed due to various clinical situations such as a patient’s agitation, operator’s experience, and any technical difficulties including a severely tortuous artery. Thus, FPR theoretically might not be quicker than multiple-pass recanalization. Considering that FPR is derived from the basis of rapid recanalization, the time to achieve FPR could be quite critical for clinical benefit from FPR. In other words, the clinical benefit of FPR can be time-dependent. However, the relationship between FPR and time to recanalization has not been reported yet. From a practical viewpoint, if one can determine the time limit to guarantee a clinical benefit from FPR, it would be so helpful to set an optimal endovascular strategy.

Accordingly, we hypothesized that favorable outcomes by FPR would be time-dependent, which meant that favorable outcomes would be not guaranteed if the time to achieve FPR was delayed. We aimed to evaluate the association between favorable outcomes and FPR under a specific time frame.

## 2. Materials and Methods

### 2.1. Study Population

We retrospectively reviewed consecutive patients with acute intracranial large vessel occlusion who underwent endovascular treatment between 2010 and 2021 in a tertiary stroke center. Endovascular treatment was generally considered for patients who met the following criteria: (1) a computed tomography (CT) angiography-determined endovascularly accessible intracranial vessel occlusion associated with neurological symptoms; (2) age ≥ 19 years; (3) baseline National Institutes of Health Stroke Scale (NIHSS) score ≥ 4; (4) time from stroke onset to groin puncture < 24 h; (5) preprocedural CT–Alberta Stroke Program Early Computed Tomography Score (CT-ASPECTS) ≥ 6; and (6) for patients with time from stroke onset > 6 h, eligibility criteria of DWI or CTP Assessment with Clinical Mismatch in the Triage of Wake-Up and Late Presenting Strokes Undergoing Neurointervention with Trevo (DAWN) and Diffusion and Perfusion Imaging Evaluation for Understanding Stroke Evolution (DEFUSE 3) trials were also considered. We preferably performed endovascular treatment in patients with a premorbid modified Rankin Scale (mRS) score ≤ 3. Patients eligible for intravenous tissue-type plasminogen activator (tPA) treatment were treated with 0.9 mg/kg tPA.

As this study focused on endovascular responses to mechanical thrombectomy, we excluded patients who had a specific occlusion etiology (e.g., arterial dissection, Moyamoya disease, etc.) and those who did not undergo a mechanical thrombectomy. Posterior circulation strokes were also excluded because clinical outcomes would be quite disparate between anterior and posterior circulation strokes. To evaluate the particular role of FPR in mechanical thrombectomy, this study included only patients with successful recanalization. The Institutional Review Board approved this study and waived the requirement of informed consent owing to the retrospective nature of this study.

### 2.2. Mechanical Thrombectomy Procedure

All endovascular procedures were performed under local anesthesia. Conscious sedation was administered when necessary. The choice between a stent retriever and a contact aspiration thrombectomy was made at the discretion of the operator. However, in most cases, a stent retriever was used as a front-line endovascular modality. An 8- or 9-F balloon guide catheter (BGC) was routinely used. A distal access catheter was hardly used. It was only used for severely tortuous arteries. A mechanical thrombectomy procedure was performed according to common recommendation [12,13]. Briefly, for stent retriever thrombectomy, a stent retriever was delivered and deployed over the thrombus using a 0.021- or 0.027-inch microcatheter. The stent retriever was left deployed for a few minutes before retrieval. For retrieval, the balloon of the BGC was inflated. The stent retriever and microcatheter were cautiously retrieved under constant aspiration using a 20 or 50 mL syringe through BGC. For contact aspiration thrombectomy, an aspiration catheter was advanced as close as possible to the proximal end of the thrombus using a coaxial technique with a microcatheter and a microwire. Contact aspiration was then performed manually using a 50 mL syringe. Concurrent contact aspiration with stent retriever thrombectomy (e.g., Solumbra, ARTS, and SAVE techniques) was not preferable. It was performed only for intractable cases. These processes were repeated until an mTICI grade of 2b or 3 was achieved. The time to discontinue attempts or switch to another endovascular modality was determined by the operator considering occlusion pathogenesis and clinical or patient condition among others.

### 2.3. Study Variables and Outcomes

Data of all variables used in this study were basically collected from a registry of patients with acute stroke. Successful recanalization was defined as a final mTICI grade 2b or 3 without further reocclusion during the procedure. FPR was defined as near-complete or complete revascularization (eTICI grade 2c or 3) after the first pass of the thrombectomy device [9,11]. For FPR, the first-pass eTICI grade 2c or 3 should be maintained without additional treatment. Two independent neurointerventionalists who were blinded to clinical information and follow-up imaging assessed study variables and outcomes including recanalization results. The κ-value for inter-rater agreement was 0.82 for successful recanalization and 0.91 for FPR. Discrepancies in the assessment of cases were resolved by consensus. Leptomeningeal collaterals were determined by CT angiography performed immediately before endovascular treatment. CT angiography collateral grade was assessed on 20 mm thickness maximum-intensity projections of single-phase images of CT angiography. Leptomeningeal collaterals were dichotomized into poor (collateral supply of ≤50% of occluded territory) and good (collateral supply of >50% of occluded territory) [14]. ASPECTS was assessed with initial non-contrast CT images [15]. When assessing leptomeningeal collaterals and ASPECTS, raters were also blinded to any endovascular information except for lesion side. The κ-value for inter-rater agreement was 0.85 for leptomeningeal collaterals and 0.66 for ASPECTS.

Functional outcome was assessed based on the mRS score at 3 months after stroke onset. Favorable outcome was defined as mRS scores of 0, 1, or 2. These mRS scores were primarily evaluated by stroke neurologists during patients’ routine clinic follow-up at 3 months (±2 weeks). If a patient could not visit the clinic, a stroke neurologist or trained nurse interviewed the patient or their family via telephone to determine the mRS score.

### 2.4. Statistical Analysis

First, to set a new recanalization status by FPR and the time from groin puncture to recanalization (puncture-to-recanalization (P-to-R) time), we tried to find a relevant P-to-R time associated with a favorable outcome. For this, we performed receiver operating characteristic (ROC) curve analysis and determined an optimal P-to-R time as the cutoff to predict favorable outcomes using the Youden index. The P-to-R time was then dichotomized based on the cutoff value. Recanalization status was categorized into four ordinal groups using a combination of FPR and the dichotomized P-to-R time. Second, to evaluate the association between the recanalization status and functional outcome, frequencies of favorable outcome were compared between four groups of recanalization status, along with a variety of clinical and endovascular variables. For trend analysis, Pearson or Spearman correlation tests and Cochran–Armitage trend tests were performed for continuous and categorical variables, respectively. To observe particular differences in P-to-R time and favorable outcome across the recanalization statuses, they were compared between the four individual groups of recanalization status using the Mann–Whitney U test and χ2 test. All *p*-values derived from multiple comparisons were adjusted by the Benjamini–Hochberg procedure. Third, to observe the independence of recanalization status for favorable outcome, multivariable logistic regression analysis was performed by adjusting for other variables with *p* < 0.1 in univariable analyses. Raw values of P-to-R time or time from stroke onset to recanalization (onset-to-recanalization (O-to-R) time) and the number of passes of the thrombectomy device were not entered into the multivariable model because they were already incorporated into the concept of recanalization status.

Statistical significance was set at *p* < 0.05 with a 95% confidence interval (CI). All statistical analyses were performed using the R software (version 4.0.1; R Foundation, https://www.r-project.org, accessed on 5 September 2023).

## 3. Results

**Figure 1:**
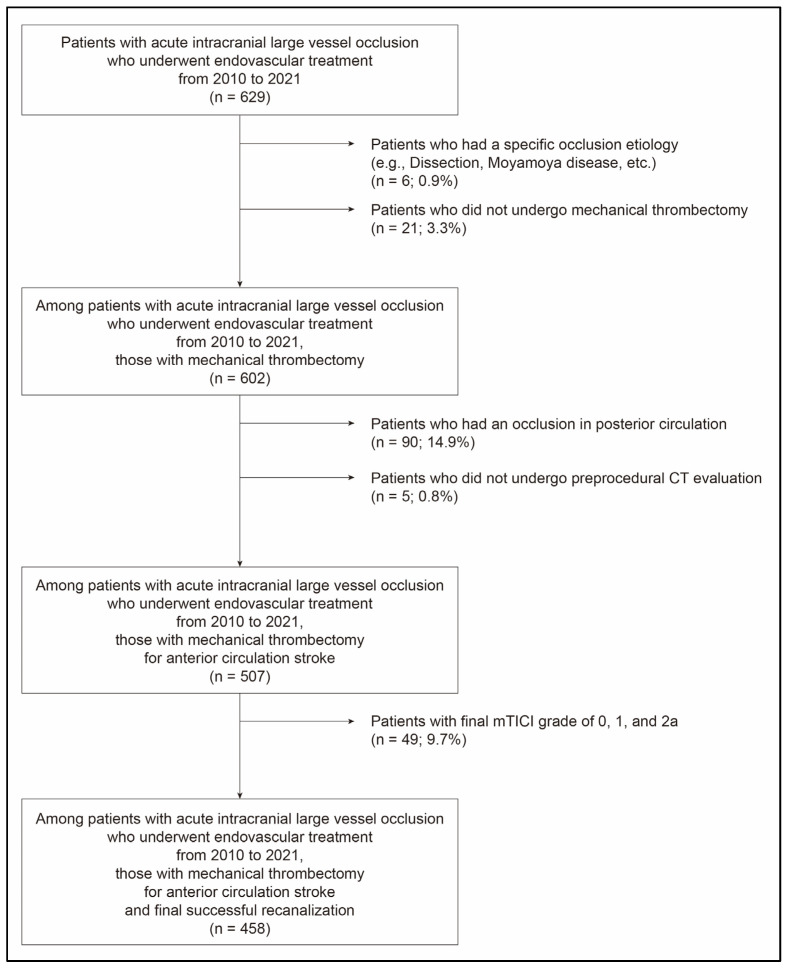
Flow chart showing selection of patients for this study. mTICI, modified Thrombolysis In Cerebral Infarction.

A total of 458 patients (mean age, 69.7 ± 12.6 years, male: 49.3%) were finally included (Figure 1). The initial NIHSS score was 15.0 (interquartile range (IQR), 11.0–19.0; Table 1) and ASPECTS was 8.0 (IQR, 6.0–9.0). A total of 291 (63.5%) patients had good leptomeningeal collaterals. Intravenous tPA was administered to 176 (38.4%) patients. Time from stroke onset to groin puncture (onset-to-puncture (O-to-P) time) was 277.0 min (IQR, 160.0–559.0 min). BGC was used in 377 (82.3%) patients. P-to-R time in the study population was 37.0 min (IQR, 22.2–63.0 min). FPR was achieved in 136 (29.7%) patients. A total of 208 (45.4%) patients had a favorable outcome.

### 3.1. Recanalization Status by FPR and P-to-R Time

In the study population, the cutoff of P-to-R time for a favorable outcome was 30.0 min (area under the ROC curve (AUC) value, 0.636 [95% CI, 0.590–0.680]; sensitivity, 51.9%; specificity, 71.2%; *p* < 0.001). Among patients with FPR, 70.6% (96 of 136) could have recanalization within 30 min (P-to-R time ≤ 30 min). However, it was 26.1% (84 of 322) for patients who did not have FPR (Table 2). Using a combination of FPR and P-to-R time, recanalization status was categorized into four groups: (1) FPR (+) with a P-to-R time ≤ 30 min (Group 1, 96 (21.0%) patients), (2) FPR (–) with a P-to-R time ≤ 30 min (Group 2, 84 (18.3%) patients), (3) FPR (+) with a P-to-R time > 30 min (Group 3, 40 (8.7%) patients), and (4) FPR (–) with a P-to-R time > 30 min (Group 4, 238 (52.0%) patients).

### 3.2. Endovascular and Functional Outcomes According to Recanalization Status

**Figure 2:**
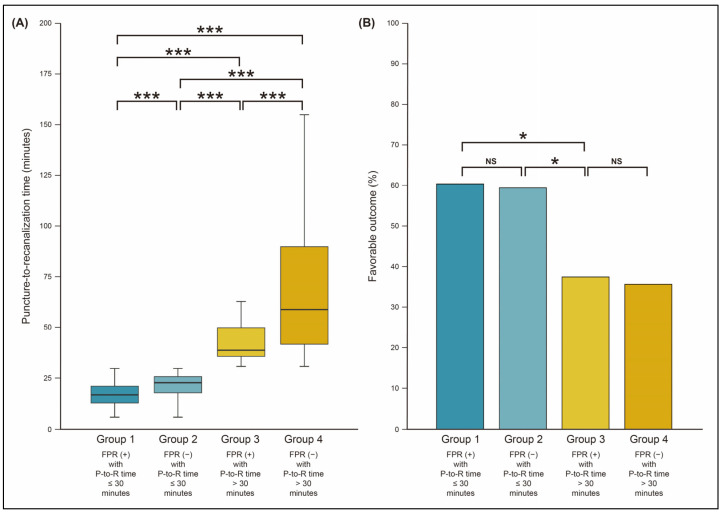
Comparison of puncture-to-recanalization (P-to-R) time (**A**) and favorable outcome (**B**) according to recanalization status by first-pass recanalization (FPR) and P-to-R time. * *p*-value < 0.05; *** *p*-value < 0.001; NS, not significant.

Among clinical findings, dyslipidemia, occlusion of middle cerebral artery, and use of BGC tended to be less frequent in patients with P-to-R time > 30 min (Group 3 and/or 4; Table 3). P-to-R time was significantly different among all groups. It tended to increase with increasing group number from Group 1 to Group 4 (*p*-value for trend < 0.001 in Table 3). P-to-R time in Group 2 (23.0 [IQR, 18.0–26.0] minutes) was significantly longer than that of the Group 1 (17.0 [IQR, 13.0–21.2] minutes; *p* < 0.001; Table 3 and Figure 2A). P-to-R time was also significantly (*p* < 0.001) different between Group 3 (39.0 [IQR, 36.0–50.0] minutes) and Group 4 (60.0 [IQR, 42.0–94.8] minutes). Likewise, O-to-R time became longer with increasing group number from Group 1 to Group 4 (*p*-value for trend < 0.001; Table 3).

**Figure 3:**
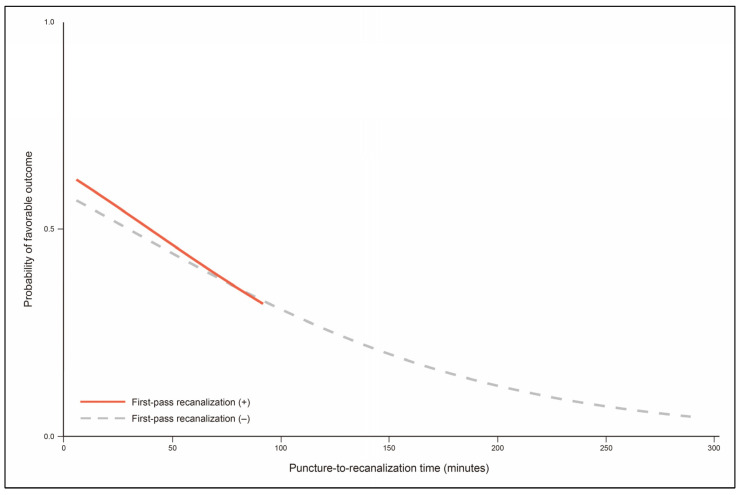
Probability of favorable outcome according to puncture-to-recanalization time. Puncture-to-recanalization time ranges from 6.0 to 92.0 min for patients with first-pass recanalization (red curve) and from 6.0 to 289.0 min for patients without first-pass recanalization (gray dashed curve).

Recanalization status was significantly associated with favorable outcome. Functional outcome was less favorable in Group 3 and Group 4 (*p*-value for trend < 0.001 in Table 3; *p* < 0.001 in Table 4). Favorable outcome was not significantly different between Group 3 (37.5%) and Group 4 (35.7%; *p* = 0.902). However, despite FPR, patients in Group 3 had less favorable functional outcomes than those in Group 2 (*p* = 0.033) and Group 1 (*p* = 0.029). In the case of FPR, probability of favorable outcome was decreased as P-to-R time increased (odds ratio, 0.82 per 10 min [95% CI, 0.65–1.04]; *p* = 0.106; Figure 3). Frequencies of patients with favorable outcomes were not significantly different between groups with a P-to-R time ≤ 30 min: 60.4% in Group 1 and 59.5% in Group 2 (*p* = 0.903; Table 3 and Figure 2B). In the multivariable analysis, recanalization status was an independent factor for favorable outcome (Table 5). Specifically, compared to Group 1, Group 2 did not show significantly altered functional outcome (adjusted odds ratio, 0.57 [95% CI, 0.28–1.17]; *p* = 0.123). However, Group 3 (adjusted odds ratio, 0.30 [95% CI, 0.12–0.76]; *p* = 0.011) and Group 4 (0.25 [95% CI, 0.14–0.48]; *p* < 0.001) were adversely associated with favorable outcome.

## 4. Discussion

In the present study, we found that recanalization status by FPR and P-to-R time was significantly associated with functional outcome after mechanical thrombectomy. It should be noted that (1) even if FPR was achieved, favorable outcome might not be guaranteed if the P-to-R time was > 30 min, such as those in Group 3; and (2) for patients who had a rapid recanalization without FPR, functional outcome might be as favorable as those with FPR if the P-to-R time was ≤ 30 min, such as those in Group 2. Accordingly, the effect of FPR on favorable outcome seems to be time-dependent. For better functional outcomes, as always, one might need to focus on the P-to-R time in addition to achieving FPR.

Beyond the traditional endovascular endpoint, FPR has recently been highlighted to achieve the best endovascular performance. The conceptual relevance of FPR has also been demonstrated in several studies as it is significantly associated with superior clinical outcomes [8,9,10,11,16]. In fact, FPR involves two distinctive points of endovascular performance: (1) near-complete or complete recanalization and (2) a single pass of a thrombectomy device. Reperfusion to mTICI grade 2b or 3 has long been regarded as a practical goal in endovascular treatment [2]. However, the degree of reperfusion varies widely from 50% to near-complete, even in one category of mTICI grade 2b, which often does not correspond to clinical outcome [3,4]. In contrast, further improved reperfusion status using eTICI grades is consistently associated with a better clinical outcome [5,6].

For practical purposes, a continuous time frame can be segmented into the number of passes of the thrombectomy device [7]. Among them, single-pass recanalization has been proposed as a strategy to shorten the time to achieve recanalization. Accordingly, FPR should be understood as a surrogate of rapid recanalization rather than simply representing the number of procedural maneuvers. For FPR, P-to-R time might be more critical than the number of passes of the thrombectomy device. First, in comparison with recanalization by multiple passes of the thrombectomy device, FPR always shows a shorter P-to-R time [9,11,17,18,19,20,21,22,23]. It means that the P-to-R time could be a significant factor for better clinical outcome even in FPR. However, unfortunately, the independence of the P-to-R time under FPR was not evaluated in previous studies. Moreover, it might be possible that not all FPRs have a P-to-R time short enough for better clinical outcomes, although the P-to-R time is statistically shorter in FPR. Actually, the upper range of the P-to-R time varies. It is commonly up to about 60 min in previous reports. Such heterogeneity of the P-to-R time in FPR can be a factor reducing the clinical benefit of FPR. Second, physical influence by multiple passes of the thrombectomy device seems to be not so significant. Experimentally, more passes of the thrombectomy device might cause arterial injury [24]. Also, such multiple passes of the thrombectomy device are associated with clot fragmentation or microembolic shower. In one in vitro study, the number of passes of the thrombectomy device was one important factor of microembolic shower based on post-procedural images [25,26]. However, such adverse events were not observed in clinical studies. Arterial injury including dissection is not significantly different between single and multiple passes of the thrombectomy device [17,21]. Considering the fact that endovascular complications by multiple passes of the thrombectomy device are not common, the harmfulness of multiple passes might not be so critical in endovascular treatment outcomes.

One interesting finding of this study was that FPR was not associated with functional outcome if only the P-to-R time was controlled by a relevant threshold. As observed, functional outcomes were comparable in early groups (between Group 1 and Group 2) and late groups (Group 3 and Group 4). In subgroup analyses, FPR and P-to-R time were not associated with favorable outcome for early groups (Supplementary Table S1) or late groups (Supplementary Table S2).

Although this study broke the common belief that FPR would be omnipotent for better clinical outcome, we still think that FPR should be achieved. Under the common practical condition, as shown in this study, more than 70% of patients with FPR could have their recanalization with a P-to-R time ≤ 30 min. Achieving a rapid FPR might be the only modifiable procedural factor for better clinical outcome. Moreover, without a time frame, lone FPR was still significantly associated with favorable outcome.

It is obvious that first-pass recanalization is a reliable and achievable goal to represent the better endovascular performance in acute stroke. However, a single pass of thrombectomy device is only a surrogate for a segmented time frame. Rapidity inherent in first-pass recanalization seems to have been overrated. This study highlighted the necessity of additional effort for more rapid recanalization even in a single pass of a thrombectomy device. In fact, an endovascular endpoint has evolved over the years to make the clinical outcome better, from simple reperfusion grade (e.g., mTICI grade 2b or 3 and eTICI grade 2c or 3) to first-pass recanalization. This study might arouse a specific condition to maximize the clinical benefit of first-pass recanalization.

This study had limitations. First, as this study was performed retrospectively, endovascular procedures were not protocolized. Many were decided at the operator’s discretion. This might have affected FPR in this study. However, endovascular procedures were performed homogeneously in a single center. We strictly followed the general common methods for mechanical thrombectomy and attempted to minimize variations, such as combining a stent retriever and an aspiration catheter. Moreover, stent retriever thrombectomy was chosen as the front-line treatment technique in most cases and Solitaire^®^ was exclusively used. Thus, FPR, a chief variable in this study, did not appear to be seriously affected by the retrospective study design.

Second, the generalizability of recanalization status derived in this study could be limited. The P-to-R time to predict favorable outcome was 30 min in this study. However, the cutoff could be different for other study populations. It would be affected by the characteristics of the study population and the eligibility of endovascular treatment. After all, a specific P-to-R time to be achieved in FPR would be changed, although the concept of rapid FPR is still valid. To utilize the study findings in real practice, the cutoff should be constant. To derive recanalization status with a constant P-to-R time and overcome the limited generalizability, multicenter-based research of a large study population is necessary. Based on the constant P-to-R time, we might be able to make a general rule in achieving proper FPR.

## 5. Conclusions

Recanalization status by FPR and P-to-R time was significantly and independently associated with functional outcome after mechanical thrombectomy. FPR was associated with functional outcome in a time-dependent manner. Even for patients who achieved FPR, their functional outcome might not be favorable if the P-to-R time was > 30 min. To secure the benefit from FPR, recanalization should be as rapid as possible even in cases with FPR.

## Figures and Tables

**Table 1:** Characteristics of the study population.

	**n = 458**
**Demographics and stroke risk factors**	
Age (years)	69.7 (±12.6)
Men	226 (49.3)
Hypertension	312 (68.1)
Diabetes	135 (29.5)
Dyslipidemia	104 (22.7)
Current smoking	70 (15.3)
Coronary artery occlusive disease	97 (21.2)
Atrial fibrillation	265 (57.9)
**Clinical conditions**	
Initial NIHSS score	15.0 [11.0; 19.0]
Intravenous tPA administration	176 (38.4)
Location of occlusion	
Internal carotid artery	173 (37.8)
Middle cerebral artery	285 (62.2)
ASPECTS	8.0 [6.0; 9.0]
Good leptomeningeal collaterals	291 (63.5)
O-to-P time (minutes)	270.0 [160.0; 559.0]
Use of balloon guide catheter	377 (82.3)
**Endovascular outcomes**	
Time to successful recanalization	
P-to-R time (minutes)	37.0 [22.2; 63.0]
O-to-R time (minutes)	341.0 [208.0; 599.0]
First-pass recanalization	136 (29.7)
Number of passes of thrombectomy device	2.3 (±1.5)
**Favorable outcome**	208 (45.4)

Values are presented as mean with standard deviation (±) or number of patients (in %). Values in brackets represent the first and third quartiles with median values. NIHSS, National Institutes of Health Stroke Scale; tPA, tissue-type plasminogen activator; ASPECTS, Alberta Stroke Program Early CT Score; O-to-P, onset-to-puncture; P-to-R, puncture-to-recanalization; O-to-R, onset-to-recanalization.

**Table 2:** Four recanalization statuses by first-pass recanalization and puncture-to-recanalization (P-to-R) time.

	First-Pass Recanalization (+) (n = 136)	First-Pass Recanalization (–) (n = 322)	Total (n = 458)
P-to-R time ≤ 30 min	96 (21.0)	84 (18.3)	180 (39.3)
P-to-R time > 30 min	40 (8.7)	238 (52.0)	278 (60.7)

Frequencies are shown in %. They were calculated as percentages of all study patients (n = 458).

**Table 3:** Clinical and endovascular findings according to recanalization status by first-pass recanalization (FPR) and puncture-to-recanalization (P-to-R) time.

	Group 1 FPR (+) with P-to-R ≤ 30 min (n = 96)	Group 2 FPR (−) with P-to-R ≤ 30 min (n = 84)	Group 3 FPR (+) with P-to-R > 30 min (n = 40)	Group 4 FPR (−) with P-to-R > 30 min (n = 238)	*p*-Value *
**Demographics and stroke risk factors**					
Age (years)	70.7 (±13.9)	65.7 (±12.8)	73.7 (±9.42)	70.0 (±12.2)	0.535
Men	46 (47.9)	40 (47.6)	20 (50.0)	120 (50.4)	0.611
Hypertension	68 (70.8)	52 (61.9)	30 (75.0)	162 (68.1)	0.993
Diabetes	33 (34.4)	20 (23.8)	18 (45.0)	64 (26.9)	0.372
Dyslipidemia	27 (28.1)	23 (27.4)	10 (25.0)	44 (18.5)	0.028
Current smoking	8 (8.3)	16 (19.0)	5 (12.5)	41 (17.2)	0.121
Coronary artery occlusive disease	17 (17.7)	12 (14.3)	14 (35.0)	54 (22.7)	0.139
Atrial fibrillation	64 (66.7)	49 (58.3)	20 (50.0)	132 (55.5)	0.079
**Clinical conditions**					
Initial NIHSS score	15.0 [12.0; 19.0]	14.0 [9.8; 18.2]	16.0 [11.0; 19.0]	16.0 [10.2; 19.0]	0.308
Intravenous tPA administration	34 (35.4)	38 (45.2)	14 (35.0)	90 (37.8)	0.914
Location of occlusion					0.028
Internal carotid artery	30 (31.2)	27 (32.1)	15 (37.5)	101 (42.4)	
Middle cerebral artery	66 (68.8)	57 (67.9)	25 (62.5)	137 (57.6)	
ASPECTS	8.0 [6.0; 9.3]	8.0 [7.0; 9.0]	8.0 [7.0; 10.0]	8.0 [6.0; 9.0]	0.199
Good leptomeningeal collaterals	60 (62.5)	53 (63.1)	22 (55.0)	156 (65.5)	0.576
O-to-P time (minutes)	226.0 [144.0; 562.0]	322.0 [155.0; 526.0]	292.0 [178.0; 508.0]	266.0 [168.0; 613.0]	0.225
Use of balloon guide catheter	90 (93.8)	81 (96.4)	26 (65.0)	180 (75.6)	<0.001
**Endovascular outcomes**					
Time to recanalization					
P-to-R time (minutes)	17.0 [13.0; 21.2]	23.0 [18.0; 26.0]	39.0 [36.0; 50.0]	60.0 [42.0; 94.8]	<0.001
O-to-R time (minutes)	245.0 [162.0; 576.0]	338.0 [174.0; 550.0]	342.0 [224.0; 546.0]	362.0 [254.0; 663.0]	<0.001
Number of passes of thrombectomy device	1.0 (± 0.0)	2.1 (± 0.84)	1.0 (± 0.0)	3.2 (± 1.5)	<0.001
**Favorable outcome**	58 (60.4)	50 (59.5)	15 (37.5)	85 (35.7)	<0.001

Values are presented as mean with standard deviation (±), median with the first and third quartiles (in brackets), or the number of patients (in %). * *p*-value for trend. NIHSS, National Institutes of Health Stroke Scale; tPA, tissue-type plasminogen activator; ASPECTS, Alberta Stroke Program Early CT Score; O-to-P, onset-to-puncture; O-to-R, onset-to-recanalization.

**Table 4:** Clinical and endovascular findings according to favorable outcome.

	Favorable Outcome (–) (n = 250)	Favorable Outcome (+) (n = 208)	*p*-Value
**Demographics and stroke risk factors**			
Age (years)	72.4 (±11.9)	66.4 (±12.7)	<0.001
Men	111 (44.4)	115 (55.3)	0.020
Hypertension	177 (70.8)	135 (64.9)	0.178
Diabetes	80 (32.0)	55 (26.4)	0.194
Dyslipidemia	47 (18.8)	57 (27.4)	0.029
Current smoking	32 (12.8)	38 (18.3)	0.105
Coronary artery occlusive disease	42 (16.8)	55 (26.4)	0.012
Atrial fibrillation	154 (61.6)	111 (53.4)	0.076
**Clinical conditions**			
Initial NIHSS score	17.0 [14.0; 20.0]	13.0 [9.0; 16.0]	<0.001
Intravenous tPA administration	84 (33.6)	92 (44.2)	0.020
Location of occlusion			0.005
Internal carotid artery	109 (43.6)	64 (30.8)	
Middle cerebral artery	141 (56.4)	144 (69.2)	
ASPECTS	7.0 [5.0; 9.0]	9.0 [8.0; 10.0]	<0.001
Good leptomeningeal collaterals	132 (52.8)	159 (76.4)	<0.001
O-to-P time (minutes)	272.0 [159.0; 645.0]	266.0 [164.0; 508.0]	0.432
Use of balloon guide catheter	195 (78.0)	182 (87.5)	0.008
**Endovascular outcomes**			
Time to successful recanalization			
P-to-R time (minutes)	42.5 [25.2; 74.0]	29.0 [19.0; 49.2]	<0.001
O-to-R time (minutes)	354.0 [222.0; 670.0]	315.0 [195.0; 547.0]	0.041
FPR	63 (25.2)	73 (35.1)	0.021
Number of passes of thrombectomy device	2.6 (± 1.6)	2.1 (±1.3)	<0.001
Recanalization status			<0.001
Group 1: FPR (+) with P-to-R time ≤ 30 min	38 (15.2)	58 (27.9)	
Group 2: FPR (–) with P-to-R time ≤ 30 min	34 (13.6)	50 (24.0)	
Group 3: FPR (+) with P-to-R time > 30 min	25 (10.0)	15 (7.2)	
Group 4: FPR (–) with P-to-R time > 30 min	153 (61.2)	85 (40.9)	

Values are presented as mean with standard deviation (±), median with the first and third quartiles (in brackets), or the number of patients (in %). NIHSS, National Institutes of Health Stroke Scale; tPA, tissue-type plasminogen activator; ASPECTS, Alberta Stroke Program Early CT Score; O-to-P, onset-to-puncture; P-to-R, puncture-to-recanalization; O-to-R, onset-to-recanalization; FPR, first-pass recanalization.

**Table 5:** Clinical and endovascular factors associated with favorable outcome.

	aOR (95% CI)	*p*-Value
Age (per year)	0.96 (0.94–0.98)	<0.001 ***
Men	1.35 (0.85–2.16)	0.202
Hypertension	0.92 (0.55–1.54)	0.740
Dyslipidemia	1.31 (0.77–2.23)	0.313
Coronary artery occlusive disease	3.10 (1.75–5.48)	<0.001 ***
Atrial fibrillation	1.31 (0.80–2.14)	0.287
Initial NIHSS score	0.90 (0.86–0.94)	<0.001 ***
Intravenous tPA administration	1.90 (1.19–3.05)	0.007 **
Occlusion of middle cerebral artery	1.22 (0.76–1.97)	0.413
ASPECTS	1.31 (1.16–1.48)	<0.001 ***
Good leptomeningeal collaterals	2.07 (1.22–3.49)	0.007 **
Use of balloon guide catheter	1.98 (1.04–3.75)	0.037 *
Recanalization status		
Group 1: FPR (+) with P-to-R time ≤ 30 min	Reference	
Group 2: FPR (–) with P-to-R time ≤ 30 min	0.57 (0.28–1.17)	0.123
Group 3: FPR (+) with P-to-R time > 30 min	0.30 (0.12–0.76)	0.011 *
Group 4: FPR (–) with P-to-R time > 30 min	0.25 (0.14–0.48)	<0.001 ***

* *p*-value < 0.05; ** *p*-value < 0.01; *** *p*-value < 0.001. aOR, adjusted odds ratio; CI, confidence interval; NIHSS, National Institutes of Health Stroke Scale; tPA, tissue-type plasminogen activator; ASPECTS, Alberta Stroke Program Early CT Score; FPR, first-pass recanalization; P-to-R, puncture-to-recanalization.

## Data Availability

The data presented in this study are available on request from the corresponding author.

## Supplementary Materials

The following supporting information can be downloaded at: https://www.mdpi.com/article/10.3390/jcm12206596/s1, Table S1: Clinical and endovascular findings according to favorable outcome in patients with puncture-to-recanalization (P-to-R) time ≤ 30 minutes. Table S2: Clinical and endovascular findings according to favorable outcome in patients with puncture-to-recanalization (P-to-R) time > 30 minutes.
