# Supplementary material for: Clinical Benefit of First-Pass Recanalization Is Time-Dependent in Endovascular Treatment of Acute Ischemic Stroke
Source: J Clin Med. 2023 Oct 18;12(20):6596. doi: 10.3390/jcm12206596 (PMC10607503; doi:10.3390/jcm12206596)
Supplement: Supplementary file 1 [file jcm-12-06596-s001.zip › jcm-2662345-supplementary.pdf]

Supplementary Materials

Table S1. Clinical and endovascular findings according to favorable outcome in patients with puncture-to-recanalization (P-to-R) time ≤ 30 minutes.

	Favorable outcome (–) (n = 72)	Favorable outcome (+) (n = 108)	*P*-value
**Demographics and stroke risk factors**			
Age (years)	71.4 (± 13.2)	66.4 (± 13.5)	0.014
Men	26 (36.1)	60 (55.6)	0.011
Hypertension	51 (70.8)	69 (63.9)	0.333
Diabetes	22 (30.6)	31 (28.7)	0.789
Dyslipidemia	20 (27.8)	30 (27.8)	0.999
Current smoking	9 (12.5)	15 (13.9)	0.788
Coronary artery occlusive disease	11 (15.3)	18 (16.7)	0.804
Atrial fibrillation	48 (66.7)	65 (60.2)	0.378
**Clinical conditions**			
Initial NIHSS score	16.5 [13.8; 20.0]	12.5 [8.0; 16.2]	<0.001
Intravenous tPA administration	25 (34.7)	47 (43.5)	0.238
Location of occlusion			0.472
Internal carotid artery	25 (34.7)	32 (29.6)	
Middle cerebral artery	47 (65.3)	76 (70.4)	
ASPECTS	7.0 [5.0; 9.0]	9.0 [8.0; 10.0]	<0.001
Good leptomeningeal collaterals	30 (41.7)	83 (76.9)	<0.001
O-to-P time (minutes)	254.0 [142.0; 604.0]	270.0 [156.0; 522.0]	0.867
Use of balloon guide catheter	68 (94.4)	103 (95.4)	0.780
**Endovascular outcomes**			
First-pass recanalization	38 (52.8)	58 (53.7)	0.903
Time to successful recanalization			
P-to-R time (minutes)	20.0 [15.8; 23.0]	19.0 [15.0; 25.0]	0.561
O-to-R time (minutes)	275.0 [162.0; 622.0]	290.0 [174.0; 538.0]	0.939
Number of passes of thrombectomy device	1.6 (± 0.9)	1.5 (± 0.8)	0.552

Values are presented as mean with standard deviation (±), median with the first and third quartiles (in brackets), or the number of patients (in %).

NIHSS, National Institutes of Health Stroke Scale; tPA, tissue-type plasminogen activator; ASPECTS, Alberta Stroke Program Early CT Score; O-to-P, onset-to-puncture; O-to-R, onset-to-recanalization.

Table S2. Clinical and endovascular findings according to favorable outcome in patients with puncture-to-recanalization (P-to-R) time > 30 minutes.

	Favorable outcome (–) (n = 178)	Favorable outcome (+) (n = 100))	*P*-value
**Demographics and stroke risk factors**			
Age (years)	72.8 (± 11.3)	66.5 (± 11.9)	<0.001
Men	85 (47.8)	55 (55.0)	0.246
Hypertension	126 (70.8)	66 (66.0)	0.407
Diabetes	58 (32.6)	24 (24.0)	0.132
Dyslipidemia	27 (15.2)	27 (27.0)	0.017
Current smoking	23 (12.9)	23 (23.0)	0.030
Coronary artery occlusive disease	31 (17.4)	37 (37.0)	<0.001
Atrial fibrillation	106 (59.6)	46 (46.0)	0.029
**Clinical conditions**			
Initial NIHSS score	17.0 [14.0; 20.0]	13.0 [9.0; 16.0]	<0.001
Intravenous tPA administration	59 (33.1)	45 (45.0)	0.049
Location of occlusion			0.014
Internal carotid artery	84 (47.2)	32 (32.0)	
Middle cerebral artery	94 (52.8)	68 (68.0)	
ASPECTS	7.0 [5.0; 9.0]	8.0 [8.0; 10.0]	<0.001
Good leptomeningeal collaterals	102 (57.3)	76 (76.0)	0.002
O-to-P time (minutes)	276.0 [167.0; 680.0]	262.0 [178.0; 496.0]	0.581
Use of balloon guide catheter	127 (71.3)	79 (79.0)	0.162
**Endovascular outcomes**			
First-pass recanalization	25 (14.0)	15 (15.0)	0.828
Time to successful recanalization			
P-to-R time (minutes)	59.0 [40.2; 94.8]	50.0 [39.0; 75.5]	0.051
O-to-R time (minutes)	374.0 [258.0; 724.0]	342.0 [224.0; 553.0]	0.123
Number of passes of thrombectomy device	3.0 (± 1.7)	2.7 (± 1.4)	0.200

Values are presented as mean with standard deviation (±), median with the first and third quartiles (in brackets), or the number of patients (in %).

NIHSS, National Institutes of Health Stroke Scale; tPA, tissue-type plasminogen activator; ASPECTS, Alberta Stroke Program Early CT Score; O-to-P, onset-to-puncture; O-to-R, onset-to-recanalization.
